# A Retrospective Study on Correlations Between EEG Signals (N20, Spectral Entropy, and Alpha Variability) and Prognosis of Traumatic Brain Injury

**DOI:** 10.3390/biomedicines14051033

**Published:** 2026-05-01

**Authors:** Xia Liu, Mengxu Qiao, Qi Liu, Meilin Ai, Jing Deng, Jian Wang, Haojun Yang, Li Huang

**Affiliations:** 1 Department of Critical Care Medicine, Xiangya Hospital, Central South University, Changsha 410008, China; lx3248352852@163.com (X.L.); 238112263@csu.edu.cn (M.Q.); lq243727710@163.com (Q.L.); aimeilin525@163.com (M.A.); djing2024@163.com (J.D.); 2 Hunan Provincial Clinical Research Center for Critical Care Medicine, Xiangya Hospital, Central South University, Changsha 410008, China; 3 National Clinical Research Center for Geriatric Disorders, Xiangya Hospital, Central South University, Changsha 410008, China; 4011378@csu.edu.cn; 4 Department of Neurosurgery, Xiangya Hospital, Central South University, Changsha 410008, China; 5 Department of Anesthesiology, Xiangya Hospital, Central South University, Changsha 410008, China

**Keywords:** traumatic brain injury, prognosis, N20, spectral entropy, alpha variability

## Abstract

**Aim:** To observe the correlations between electroencephalography (EEG) signals and clinical outcomes in patients with traumatic brain injury (TBI). **Methods:** A total of 174 patients diagnosed with TBI at Xiangya Hospital during January 2017 and June 2024 were included in this study. Quantitative EEG parameters, including spectral entropy (SE), alpha variability (RAV), and relative spectral energy (RBP), along with somatosensory evoked potential (SSEP) recordings (N20 amplitude) were assessed within 7–14 days after the disease onset. Patients were divided into a good-prognosis group and a poor-prognosis group based on the Glasgow Outcome Scale (GOS) scores at six months after discharge. **Results:** Significant correlations were found between the initial Synek EEG grading and 6-month GOS score (ρ = −0.709, *p* < 0.001). Compared with patients in the poor-prognosis group, significantly higher N20 amplitudes (*p* < 0.001), higher SE (*p* = 0.049), higher RAV (*p* = 0.009), and lower relative beta energy (*p* < 0.05) were found in TBI patients with good prognosis. Among these parameters, N20 amplitude demonstrated the best predictive performance. The N20 amplitude threshold of >1.975 μV predicted a good outcome with a sensitivity of 93.3% and a specificity of 94.1%. **Conclusions:** These findings may provide a reliable and sensitivity method to evaluate and predict the prognosis of TBI patients, which has important clinical management significance.

## 1. Introduction

Traumatic brain injury (TBI) is a major public health challenge and has become the leading cause of disability and death in all types of traumatic injuries worldwide [1]. Approximately 50 million people suffer from TBI each year globally, with an estimated economic burden of $400 billion annually [2,3]. And the number of patients with TBI in China far exceeds that in most other countries [4]. Among these cases, approximately 80% cases are classified as mild, while the remaining 20% are moderate to severe. Moderate to severe TBI is often associated with consciousness disorders, cognitive impairment, psychosocial deficits, disability and even high mortality, exerting devastating effects on patients and imposing a substantial burden on families and society [5]. Consequently, research on TBI-related damage has increasingly attracted public attention.

Although substantial efforts have been made in the prevention and treatment of TBI, the progress in improving patient outcomes remains far from satisfactory [1]. An effective prognosis assessment is crucial for alleviating anxiety, and may even influence decisions regarding the continuation of treatment. However, the pathophysiological mechanisms following TBI are complex and it remains poorly understood. Although MRI can identify key brain regions affected by the disease, the high cost and strict conditions preclude its routine examination [6]. Several factors have been shown to correlate with TBI prognosis, including the Glasgow Coma Scale (GCS) and laboratory parameters such as circulating inflammatory proteins [7], neutrophil-to-lymphocyte ratio [8], and glycemic variability [9], yet these factors have received insufficient attention. Moreover, although several prediction models have been developed to estimate prognosis and mortality in TBI patients, their sensitivity and specificity have not gained widespread acceptance in clinical practice. Thus, accurately predicting the prognosis of TBI remains a significant challenge.

EEG are formed when millions of neurons’ postsynaptic potentials combine, reflecting the brain function. However, interpreting raw EEG signals requires extensive effort, many factors such as electrical interference signals from machines and subject’s physiological activities, like eye movement or facial muscle contractions, can introduce EEG artifacts [10,11]. Quantitative EEG (qEEG) can remove noise and artifacts that may interfere with accurate interpretation through standard band-pass filtering, thereby isolating signal components related to the physiological activity under investigation [12]. Consequently, qEEG represents an efficient monitoring tool for assessing the degree of encephalopathy [13,14,15]. Another form of EEG monitoring, short-latency somatosensory evoked potential (SSEP), has also been widely used in prognostic studies of neurological diseases in recent years, due to its noninvasive, objective, inexpensive and unique role [16,17,18]. But few studies focused on the correlation of EEG signals and the prognosis of patients with TBI. Previous studies have shown the amplitudes of N20, spectral entropy, alpha variability and relative spectrum energy are correlated with the prognosis of comatose patients. Therefore, this study aims to compare the differences in the above EEG signals between TBI patients with good and poor prognosis in intensive care unit (ICU).

The key contributions of this study can be summarized as follows:

Novelty: This study quantitatively evaluates the predictive value of N20 in TBI patients.

Methodological advancement: We applied qEEG in combination with SSEP to achieve an objective electrophysiological assessment in TBI patients.

Practical significance: Electrophysiological monitoring may provide a reliable and sensitive tool for evaluating prognosis in TBI patients, offering important guidance for clinical management.

The remainder of this paper is divided into the following sections. Section 2 presents the materials and methods, including study population and procedure, data monitoring, outcome assessment and statistical analyses. Section 3 reports the results, comparing the demographic data and EEG Signals based on prognostic differences. Section 4 provides a discussion of the findings, including points out the limitations. Finally, Section 5 concludes the paper and highlights the clinical implications of this research.

## 2. Materials and Methods

### 2.1. Study Population

All patients with TBI admitted to the Department of Critical Care Medicine, Xiangya Hospital, Central South University from January 2017 to June 2024 were enrolled in this study. The inclusion criteria were as follows: (1) a clear history of TBI; (2) aged between 18 and 80 years; and (3) admission within 7–14 days after injury onset. The exclusion criteria were as follows: (1) known history of neurological disorders, such as stroke, tumor, or dementia, etc.; (2) trauma following or combined with respiratory or cardiac arrest, (3) definite peripheral neuropathy or spinal cord injury, (4) death due to non-neurological complications, such as severe infection, and (5) loss to follow-up.

The sample size was calculated by the formula N = Z^2^ × (P × (1 − P))/E^2^. With a confidence level of 90% (Z value is 1.64), an error value (E) of 10%, and a probability value (P) of 0.5, the calculated sample size (N) was 67.

### 2.2. Study Procedure

This was a retrospective study, baseline characteristics were collected including demographics (i.e., age, sex, disease type, and length of ICU stay). The qEEG and SSEP recordings were obtained within 7–14 days after disease onset. When multiple recordings were available, the average values were used for analysis. To minimize potential interference, the cumulative dose of sedatives and analgesics administered prior to neurophysiological monitoring were also recorded and excluded. At six months after discharge, the patients were followed up via telephone and reassessed using the Glasgow Outcome Scale (GOS).

All neurophysiological recordings were interpreted by two specially trained ICU physicians, who were blinded to the clinical conditions and patient outcomes during both data collection and statistical analysis.

### 2.3. SSEP Monitering

SSEP was recorded using a Nicolet evoked potential machine. The median nerves were alternately stimulated at each wrist with an intensity sufficient to evoke a visible thenar muscle twitch. The recording electrodes were placed according to the international EEG 10–20 system. The locations of the recording electrode pairs were (1) C’3 or C’4: 2 cm posterior to C3 and C4, overlying the contra-lateral somatosensory cortex; (2) Fz: the midpoint of the forehead; (3) Cv: located at the 7th cervical vertebra; and (4) bilateral Erb’s points: 1 cm above the midpoint of the contralateral clavicle, with the ipsilateral point designated as CLi and the contralateral point as CLc. The ground electrode was placed 5 cm above the stimulation site, and the reference electrode was placed at FPz, the midpoint of the frontal pole. The electrode impedance was kept at 5000 ohms, and the recording band-pass filter was set between 3 Hz and 2000 Hz. The analysis time was 50 ms, stimulus pulse duration was 0.1–0.2 ms, and the stimulus rate was 1–5 Hz. Each measurement was recommended to be repeated at least twice to obtain an averaged response, with each repetition comprising a minimum of 500 stimulus presentations.

**Figure 1:**
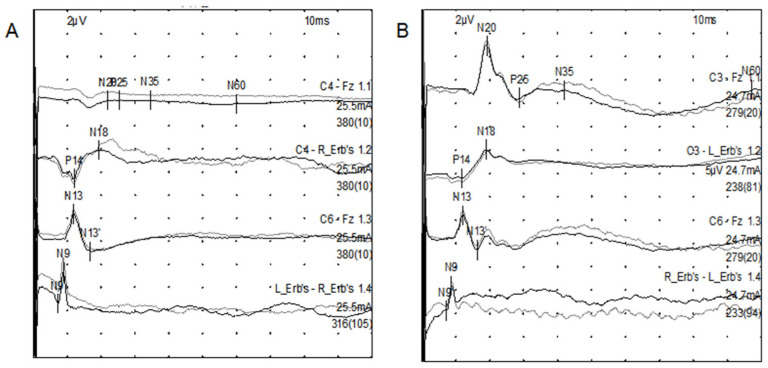
The amplitudes of N20. Time is plotted on the *X*-axis with a scale interval of 10 ms, and amplitude is plotted on the *Y*-axis with a scale interval of 2 uV. (**A**) shows that the N20 amplitude was absent, indicating severe brain injury, (**B**) shows that the N20 amplitude is within the normal range, indicating mild brain injury.

The N20 amplitude was defined as the difference between the upwards negative wave peak and the subsequent downwards negative wave peak (i.e., the N20–P25 peak-to-peak amplitude) approximately 20 ms after stimulation. The final amplitude was calculated as the average amplitude of both sides (Figure 1).

### 2.4. QEEG Monitering

The Nicolet One Monitor analyzes collected signals using Fast Fourier Transform (FFT) and employs quantitative analysis techniques for frequency-domain analysis. qEEG recordings were obtained using a Nicolet system within 14 days of disease onset. The electrodes were placed according to the international 10–20 system, with 8 channels (Fp1, Fp2, C3, C4, T3, T4, O1, and O2). Cz served as the reference electrode, and Fpz served as the ground. The filter channel was set to 1–35 Hz, the sample frequency to 125 Hz, and time constant to 0.3 s.

#### 2.4.1. Relative Band Power (RBP)

EEG background activity is categorized into different frequency bands, including delta (0.4–4 Hz), theta (4–8 Hz), alpha (8–12 Hz), and beta (13–30 Hz) [19]. RBP data were calculated by determining the proportion of EEG activities at different frequencies within every 10 s, thereby obtaining the frequency-band energy area, which refers to the proportion of energy in each band power to total frequency power.

#### 2.4.2. Relative Alpha Variability (RAV)

RAV reflects the variability of proportion that 6–14 Hz power within the total frequency power activity (RA). The RAV was automatically calculated every 2 min using the formula: (peak RA − trough RA)/(peak RA + trough RA), with higher values indicating better cerebral blood flow and oxygen metabolism.

#### 2.4.3. Spectral Entropy (SE)

SE represents the complexity of the EEG power spectrum; nonlinear dynamic methods are employed to describe the complexity, irregularity, and chaotic behavior of EEG time series. A more regular signal corresponds to a lower entropy value and indicates a deeper degree of coma. SE values were calculated every 10 s.

**Figure 2:**
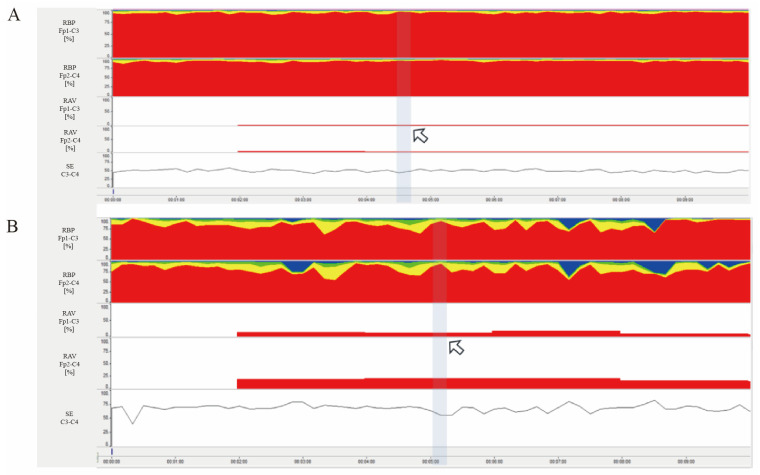
Trend chart. This trend chart presents a ten-minute interval. Time is plotted on the *X*-axis with a scale interval of 1 min, and the *Y*-axis (from top to bottom) displays RBP, RAV, and SE, respectively. RBP and RAV are expressed as percentages (%). Arrows mark the calibration points, from which precise values can be obtained at any moment. (**A**) illustrates severe brain injury, characterized by RBP-dominated slow-wave activity (red). In contrast, (**B**) represents mild brain injury, where RBP shows prominent fast-wave activity (yellow and blue). Additionally, compared to (**B**), both RAV and SE values in (**A**) are relatively lower.

All patients were monitored on qEEG for at least 12 h, EEG data were then exported digitally from a computer hard drive. The raw EEG waveforms were reviewed to exclude artifact-based data that may have contaminated the trends. Finally, the mean values of RBP, RAV and SE for each montage were calculated (Figure 2).

### 2.5. EEG Grading

Raw EEG waveforms were graded according to the Synek scale. Grade 1: abnormality with alpha activity but little, scattered theta activity. Grade 2: abnormality with predominant theta activity; one subgrade is reactive; the second subgrade is nonreactive. Grade 3: abnormality with continuous rhythmic delta activity; one subgrade is large-amplitude rhythmic delta activity, a second subgrade is spindle coma pattern, another subgrade is small-amplitude, diffuse irregular delta activity, the other one is widespread moderate-amplitude delta activity. Grade 4: abnormality with frequent isoelectric intervals; one subgrade is epileptiform discharges, a second subgrade is alpha pattern coma, a third subgrade is theta pattern coma, a fourth subgrade in Grade 4 is low-output EEG. Grade 5: abnormality with isoelectric EEG.

### 2.6. Outcome Assessment

Patient outcomes were assessed at six months after discharge using the GOS, which comprises five outcome categories: 1, dead; 2, vegetative state; 3, severe cerebral disability; 4, moderate cerebral disability; and 5, good cerebral recovery. The scores 3 to 5 were defined as awakened with a favorable prognosis, whereas scores 1 and 2 were defined as unawakened with a poor prognosis.

### 2.7. Statistical Analyses

All statistical analyses were performed using SPSS software (22.0). Categorical variables were expressed as counts and percentages, and the chi-square test was used for comparison between the two outcome groups. Continuous variables conforming to a normal distribution were presented as mean and standard deviation, and differences between groups were assessed using the *t*-test. For continuous variables that were not normally distributed, the Mann–Whitney U test was applied, with data described as median (interquartile range). Spearman’s rank correlation analysis was used for ordinal variables. A *p*-value < 0.05 was considered statistically significant. Receiver operating characteristic (ROC) curve analysis was employed to evaluate the predictive performance of each indicator for neurological outcomes, and the area under the curve (AUC) was calculated to assess sensitivity and specificity.

## 3. Results

### 3.1. Demographic Data

**Figure 3:**
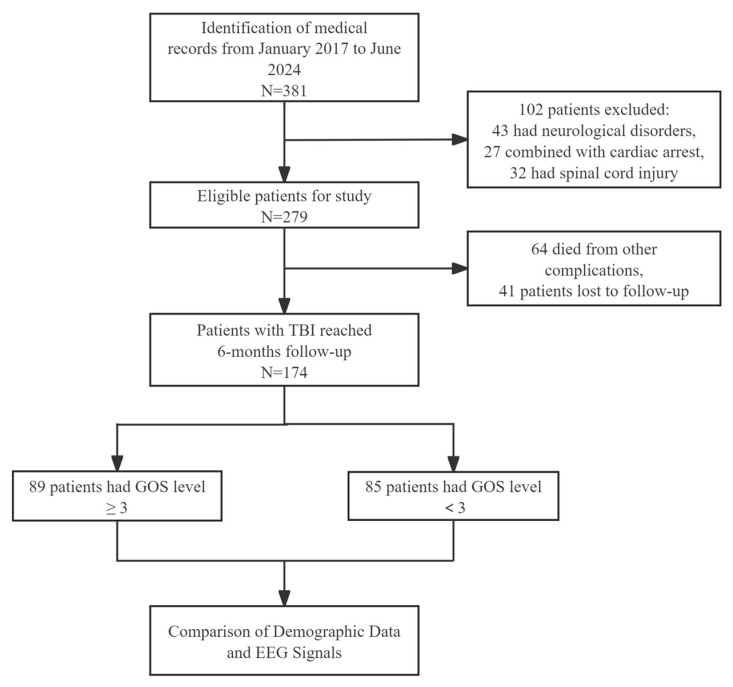
Overall framework diagram.

A total of 381 patients with TBI were identified within the study period between January 2017 to June 2024. Following the application of exclusion criteria, 174 patients were included in the final analysis (Figure 3). Patients were categorized into two groups according to their GOS scores at six months: group A (good prognosis, n = 89) and group B (poor prognosis, n = 85). There were no significant differences between the two groups with respect to demographic characteristics, including age, gender and duration of hospitalization (Table 1).

### 3.2. The Correlation Between Synek EEG Grading and GOS Score (6 Months Later)

Significant correlations (r = −0.709, *p* < 0.001) were found between the above two indicators by Spearman’s correlation analysis, suggesting EEG signals may serve as an important indicator for predicting the prognosis of TBI.

### 3.3. Amplitudes of N20

**Figure 4:**
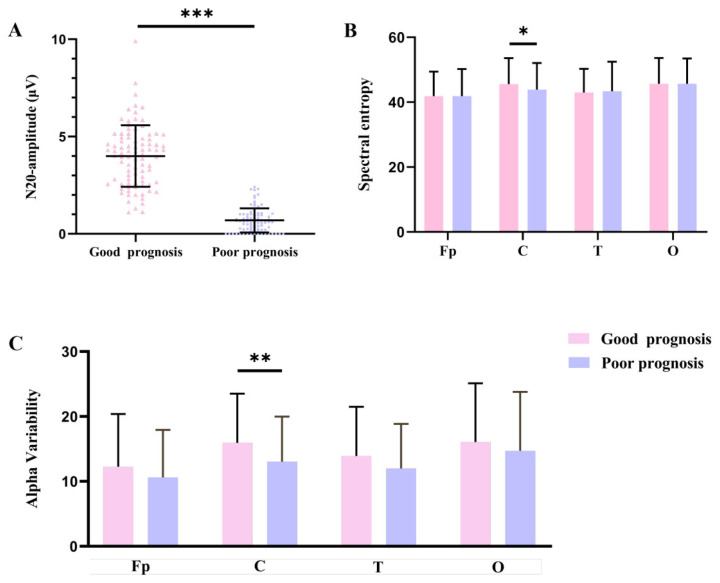
Differences in amplitudes of N20 (**A**), SE (**B**) and RAV (**C**) among TBI patients with good prognosis and poor prognosis. Notes: *** *p* value is <0.001; ** *p* value is <0.01; * *p* value is <0.05. Abbreviations: Fp means frontal-parietal electrodes; C means central electrodes; T means temporal electrodes; O means occipital electrodes.

As shown in Figure 4A, the amplitudes of N20 in the good-prognosis group (4.00 ± 1.58) were significantly higher than the poor-prognosis group’s (0.69 ± 0.62, *p* < 0.001).

### 3.4. SE

Compared with patients in the poor-prognosis group (43.89 ± 8.15), those in the good-prognosis group exhibited significantly higher SE values at central electrodes (45.55 ± 8.02). No significant differences were observed between the two groups at frontal-parietal, temporal or occipital electrodes among two groups (Figure 4B). Detailed values for each individual electrode are provided in Supplementary Table S1.

### 3.5. RAV

RAV at central electrodes was obviously higher in the good-prognosis group (15.94 ± 7.57) than in the poor-prognosis group (13.03 ± 6.96, *p* = 0.009). No significant differences were observed between the two groups at frontal-parietal, temporal, or occipital electrodes (Figure 4C). Detailed values for each individual electrode are provided in Supplementary Table S2.

### 3.6. RBP

**Figure 5:**
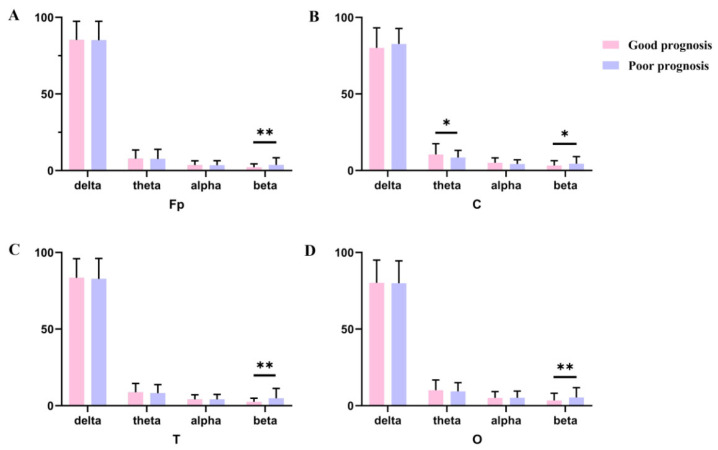
RBP (delta, theta, alpha and beta) in different brain regions ((**A**) means frontopolar electrodes, (**B**) means central electrodes, (**C**) means temporal electrodes and (**D**) means occipital electrodes). Notes: ** *p* value is <0.01; * *p* value is <0.05. Abbreviations: Fp means frontal-parietal electrodes; C means central electrodes; T means temporal electrodes; O means occipital electrodes.

In the frontal-parietal regions, relative beta energy was significantly lower in the good-prognosis group (2.18 ± 2.17) than in the poor-prognosis group (3.73 ± 4.61) (Figure 5A). At the central electrodes, patients with good prognosis exhibited significantly lower beta power (3.26 ± 3.17 vs. 4.48 ± 4.67, *p* = 0.047) and significantly higher theta power (10.53 ± 6.99 vs. 8.52 ± 4.71, *p* = 0.047) compared with those in the poor-prognosis group (Figure 5B). At temporal electrodes, the relative beta energy was significantly higher in the poor-prognosis group (4.90 ± 6.38) than in the good-prognosis group (2.53 ± 2.30) (Figure 5C). As shown in Figure 5D, patients in the good-prognosis group had significantly lower beta power at occipital electrodes (3.46 ± 4.63) compared with those in the poor-prognosis group (5.35 ± 6.43, *p* = 0.028).

### 3.7. Amplitudes of N20 Predicting a Good Outcome

**Figure 6:**
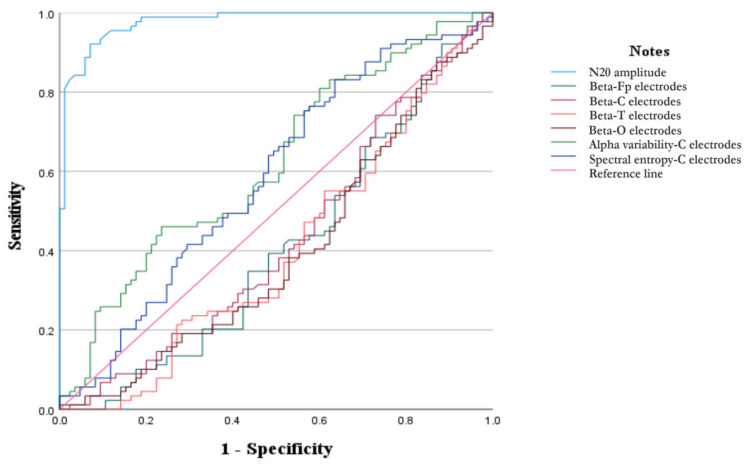
The ROC curve for TBI at 6 months showing the predictive powers of EEG signals.

ROC curves were constructed to evaluate the predictive value of the above signals for TBI outcomes. As shown in Figure 6, N20 amplitude demonstrated the best predictive performance (AUC = 0.977, *p* < 0.001), followed by RAV (AUC = 0.620, *p* = 0.042) and SE (AUC = 0.585, *p* = 0.044). In addition, an N20 amplitude threshold of >1.975 μV predicted a favorable outcome with a sensitivity of 93.3% and a specificity of 94.1%. RAV > 16.995 yielded a sensitivity of 46.1% and a specificity of 76.5% for predicting good outcomes, while SE >39.470 achieved a sensitivity of 83.1% and a specificity of 36.5%.

## 4. Discussion

This retrospective study, conducted at Xiangya Hospital, Central South University, aimed to observe the correlations between EEG signals and clinical outcomes in patients with TBI. Compared with patients in the poor-prognosis group, those with a good prognosis exhibited significantly higher N20 amplitudes, higher SE and RAV (at C3 and C4), and lower relative beta energy (at Fp1, Fp2, C3, C4, T3, T4, O1, and O2). Among these parameters, N20 amplitude demonstrated the best predictive performance, with a threshold of >1.975 μV yielding a sensitivity of 93.3% and a specificity of 94.1% for predicting a favorable outcome.

SSEP reflects the integrity of the neural pathway from peripheral sensory nerves to the cortical projection [20]. Previous studies have found the unique value of SSEPs in the prognosis and monitoring of various neurological diseases, including TBI, spinal cord injury [21] and hypoxic–ischemic encephalopathy [22], etc. In addition, several studies have demonstrated the prognostic utility of SSEPs in cardiac arrest. For example, the amplitude <0.4 μV has been consistently associated with poor outcomes of patients with cardiac arrest [23], while the amplitudes of N20-b and N20–P25 have been shown to predict both good and poor outcomes in comatose patients after cardiac arrest [22], with high specificity but low to moderate sensitivity. Similar to previous findings, the present study further confirms the predictive value of N20 amplitude in TBI, achieving both sensitivity and specificity exceeding 90%. By quantifying the N20 amplitude for the first time, this study demonstrates its value in predicting the prognosis of patients with TBI. The results indicate that lower N20 amplitudes are significantly associated with a higher probability of poor prognosis, suggesting that N20 serves as a robust predictor of six-month outcomes in TBI patients. Notably, apart from prognosis, SSEP could also predict cognitive functions, such as memory, attention and concentration. These findings all support the potential value of SSEP, especially N20, in predicting the prognosis and cognition of multiple disorders in the future. Investigations could further explore the role of N20 in predicting cognitive function in TBI patients.

EEG monitoring has been established as a sensitive and specific prognostic tool that can indicate outcomes in patients with moderate to severe TBI within 3 days post-injury [24], which to some extent supports the reliability of our findings. Although the correlation and predictive effect of SE and RAV with TBI were inferior to that of N20 amplitude in the present study, these two parameters could still reflect good predictive value in other diseases. SE is an indicator used to quantify the complexity of EEG signals. In awake individuals, brain activity is irregular and complex, which corresponds to high SE values. Conversely, when the brain sustains varying degrees of trauma and the cerebral cortex becomes suppressed, brain activity becomes relatively regular and orderly, leading to decreased SE values. Previous studies have shown that SE effectively distinguishes between conscious and unconscious states during sevoflurane, propofol and thiopental anesthesia [25], suggesting its potential as a monitor of sedation depth [26]. In our study, high SE were observed in TBI patients with good prognosis, indicating that prognosis may be predicted based on the evaluation of consciousness status in the early stage of TBI occurrence. Regarding RAV, previous studies have demonstrated its ability to distinguish between favorable and unfavorable 6-month outcomes in patients with TBI. Research on subarachnoid hemorrhage has also shown that RAV enables early detection of delayed cerebral ischemia [14]. A decline in RAV values reflects reduced focal cerebral blood flow and allows detection of ischemia-induced vasospasm 7 h before the onset of clinical symptoms and 44 h before diagnosis by computed tomography, highlighting the sensitivity of EEG signals. Multivariate modeling analysis has further revealed that diffuse edema, along with injuries to the basal ganglia and thalamus, are core anatomical predictors of RAV, whereas brainstem injury and diffuse cerebral trauma, such as multiple subcortical lesions and deep white matter shearing, were key determinants of six-month recovery [27]. These findings provide additional anatomical evidence supporting the clinical significance of RAV. However, in the current study, we did not stratify patients by injury locations to evaluate whether lesions in specific cerebral locations would affect the predictive sensitivity and specificity of N20 amplitudes or RAV with respect to patients’ outcomes.

Generally, Beta waves are associated with attention, wakefulness, alertness and cognition. For instance, patients with temporal lobe epilepsy have been shown to exhibit significantly prolonged reaction times during attention network tests compared with healthy controls, which was found to be related to reduced relative beta energy. Similarly, executive attention impairments in patients with TBI have been associated with suppression of frontal beta power [28]. In addition, a retrospective study of comatose patients with TBI reported that those who recovered had lower delta power and increased beta power [29]. However, in this study, we found that after adjusting for sedatives on outcomes, TBI patients with good prognosis still exhibited lower beta power, which is contrary to previous findings. We attribute this difference to a higher susceptibility to epileptic complications and abnormal neuronal firing in patients with poor prognosis, which may result in excessive beta energy.

Numerous studies have demonstrated that the mutual information across different frequency bands in EEG signals reveal the nonlinear coupling relationships between brain regions [30,31]. These findings highlight the need for future research to explore the correlation between EEG signal mutual information dynamics and the severity of regional brain injury. Previous studies have found that the sensitivity of using single-channel EEG for detecting epileptic seizures is only 40% [32]. Accumulating evidence indicates that predictive models incorporating both inter-channel spatial interactions and temporal dynamics enable accurate prediction of epileptic seizures [33]. Given that TBI prognosis depends on a variety of physiological, neurological, and systemic factors, future research could build predictive models by integrating multiple parameters, such as GCS scores, biomarkers, and EEG indicators to achieve greater predictive accuracy. Notably, a recent study demonstrated that functional near-infrared spectroscopy can predict cognitive dysfunction in stroke survivors by measuring hemodynamic responses in the frontotemporal cortex [34]. Another study further quantitatively assessed the reliability of functional near-infrared spectroscopy [35]. These findings offer important implications for the present study, suggesting the integration of this technique into future research.

Several limitations of this study should be acknowledged. First, this study was a single-center retrospective study with a relatively small sample size, which may limit the generalizability of the findings. Second, the number of electrodes used for continuous EEG monitoring was relatively small, making it impossible to conduct correlation analysis between brain network changes and TBI prognosis. Third, we did not stratify patients by injury locations to evaluate whether lesions in specific cerebral locations would affect the predictive sensitivity and specificity of N20 amplitudes with respect to patient outcomes.

## 5. Conclusions

Our study aimed to observe the correlations between EEG signals and outcomes of patients with TBI. We found that N20 amplitudes can predict outcomes of TBI patients at 6 months. We will further expand the sample size and integrate advanced EEG monitoring with computational modeling to conduct detailed subgroup analyses based on lesion location.

## Figures and Tables

**Table 1:** Demographic characteristics of the study population.

Items		Group A	Group B	*p* Value
N		89	85	
Age (year)		53 (43–62)	55 (34–68)	0.443
Gender (n)	Male	68	72	0.650
	Female	21	13	
Duration of hospitalization (day)		20.88 ± 11.95	20.29 ± 16.77	0.792

## Data Availability

The data presented in this study are available on request from the corresponding author.

## Supplementary Materials

The following supporting information can be downloaded at: https://www.mdpi.com/article/10.3390/biomedicines14051033/s1, Table S1: Spectral entropy in different electrodes among patients with good and poor prognosis; Table S2: Alpha variability in different electrodes among patients with good and poor prognosis.
