# Supplementary material for: A Retrospective Study on Correlations Between EEG Signals (N20, Spectral Entropy, and Alpha Variability) and Prognosis of Traumatic Brain Injury
Source: Biomedicines. 2026 May 1;14(5):1033. doi: 10.3390/biomedicines14051033 (PMC13203879; doi:10.3390/biomedicines14051033)
Supplement: Supplementary file 1 [file biomedicines-14-01033-s001.zip › biomedicines-4173291-supplementary.pdf]

Table S1. Spectral entropy in different electrodes among patients with good and poor prognosis.

Electrodes	Good prognosis	Poor prognosis	*p* value
Fp1	41.79 ± 6.92	41.27 ± 7.59	0.365
Fp2	41.99 ± 7.26	42.48 ± 9.27	0.810
C3	45.50 ± 7.50	43.96 ± 7.66	0.107
C4	45.61 ± 7.21	43.83 ± 7.94	**0.034**
T3	42.47 ± 6.83	43.75 ± 8.65	0.563
T4	43.43 ± 6.64	43.03 ± 9.11	0.219
O1	45.60 ± 7.17	45.96 ± 7.95	0.882
O2	45.73 ± 7.61	44.76 ± 7.07	0.312

Notes: Significant values were in bold.

Table S2. Alpha variability in different electrodes among patients with good and poor prognosis.

Electrodes	Good prognosis	Poor prognosis	*p* value
Fp1	12.04 ± 8.38	10.38 ± 6.98	0.158
Fp2	12.47 ± 8.48	10.84 ± 8.03	0.194
C3	15.74 ± 8.02	13.23 ± 7.46	**0.034**
C4	16.15 ± 7.71	12.83 ± 6.77	**0.003**
T3	13.32 ± 7.76	12.47 ± 8.12	0.481
T4	14.49 ± 7.86	11.48 ± 6.22	**0.006**
O1	16.21 ± 9.20	15.63 ± 10.15	0.695
O2	15.90 ± 9.24	13.81 ± 8.65	0.126

Notes: Significant values were in bold.
